# Hot Deformation Behavior via Isothermal Compression and Constitutive Model of GH2132 Superalloy

**DOI:** 10.3390/ma18245650

**Published:** 2025-12-16

**Authors:** Yue Sun, Peng Cheng, Decheng Wang, Chenxi Shao, Lu Cheng

**Affiliations:** 1 China Productivity Center for Machinery, China Academy of Machinery Science and Technology, Beijing 100044, China; sunyue97@hnu.edu.cn (Y.S.);; 2 College of Mechanical and Vehicle Engineering, Hunan University, Changsha 410082, China

**Keywords:** GH2132 superalloy, hot deformation, constitutive modeling, processing map

## Abstract

GH2132, an Ni–Cr–Fe-based superalloy for aero-engine components, exhibits hot workability that is highly sensitive to processing parameters. The hot deformation behavior of GH2132 alloy was investigated via isothermal compression (Gleeble-3500-GTC) over 850–1100 °C and 0.001–10 s^−1^, combined with optical microscopy and EBSD characterization. A strain-compensated Arrhenius-type hyperbolic-sine model was established, achieving high predictive accuracy (R^2^ = 0.9916; AARE = 3.86%) with an average activation energy Q = 446.2 kJ·mol^−1^. Flow stress decreases with increasing temperature and increases with strain rate, while microstructural softening transitions from dynamic recovery to complete dynamic recrystallization at higher temperatures and lower strain rates. Three-dimensional power-dissipation and hot-processing maps (Dynamic Materials Model) delineate safe domains and instability regions, identifying an optimal window of 1000–1100 °C at 0.001–0.01 s^−1^ and instability at 850–900 °C with 0.01–0.1 s^−1^. These results provide guidance for selecting parameters for hot deformation behavior during thermomechanical processing of GH2132.

## 1. Introduction

Ni–Cr–Fe-based superalloys are critical high-temperature structural materials, exhibiting exceptional thermal strength together with superior resistance to creep, fatigue, oxidation, and corrosion [1,2,3,4]. GH2132 is an Ni–Cr–Fe-based superalloy extensively used in aerospace applications. Owing to its high yield strength, excellent fatigue performance, and outstanding creep resistance up to approximately 650 °C, GH2132 is regarded as a prime candidate for critical aero-engine components, including turbine and compressor disks, rotor blades, and fasteners [5,6,7]. A rigorous understanding of its microstructural characteristics and mechanical behavior is essential for guiding the design and manufacture of high-performance components operating under elevated temperature and stress. However, the complex chemistry and high deformation resistance of GH2132 make it highly sensitive to processing parameters during hot working. Consequently, suboptimal deformation temperatures or strain rates can lead to undesirable microstructures and dimensional deviations [8,9]. Therefore, a systematic investigation of the hot deformation behavior and microstructural evolution of GH2132 is of considerable theoretical and practical significance for optimizing its thermomechanical processing and improving the quality of final components.

At present, the thermal deformation behavior of metallic materials is commonly analyzed through constitutive equations that quantitatively relate flow stress to strain, strain rate, and temperature, thereby offering a theoretical basis for elucidating hot deformation mechanisms and optimizing processing windows [10,11,12]. The hot deformation behavior of GH2132 alloy in cast billets subjected to solution and aging treatment—corresponding to the material state in this study—remains insufficiently explored. This state, featuring distinct grain morphology, precipitate distribution, and grain boundary characteristics, can uniquely influence plastic flow and microstructural evolution, making the establishment of a reliable constitutive model essential for optimizing forging parameters and improving formability. Therefore, investigating the hot deformation behavior and constructing a reliable constitutive model based on thermal simulation tests are essential for determining suitable forging parameters and improving the formability of the GH2132 superalloy.

Although extensive investigations have been conducted on various Ni-based superalloys, systematic studies on GH2132, particularly in the solution-aged condition originating from cast billets, remain limited. This material state contains inherited segregation features, heterogeneous precipitate distributions, and grain-boundary characteristics distinct from wrought products [13], which can markedly alter deformation resistance and the activation of softening mechanisms [14]. Furthermore, the response of GH2132 under a broad strain-rate domain—especially rates approaching industrial forging conditions—has not been comprehensively clarified [15], and the associated dynamic recrystallization behavior and flow-stability characteristics lack quantitative description [16]. The absence of such fundamental knowledge restricts the establishment of reliable constitutive relations and impedes the determination of robust hot-working windows. Accordingly, a detailed characterization of the high-temperature deformation behavior of GH2132 across an extended processing domain is required to support the optimization of thermomechanical schedules and ensure stable forming performance. Zhong et al. [17] investigated the hot deformation behavior of GH4738 alloy. A strain-compensated Arrhenius model and processing maps were developed, revealing that flow stress increased with strain rate and decreased with temperature. DDRX dominated stable regions, and optimal hot-working windows were identified at 1010–1150 °C/0.01–1 s^−1^. Wang et al. [18] examined the hot deformation behavior of GH3128 alloy via hot tensile tests at 750–950 °C. EBSD and DSC analyses revealed twin- and particle-stimulated dynamic recrystallization. M_23_C_6_ and MC carbides precipitated with increasing temperature, leading to grain coarsening and a ductile-to-brittle fracture transition. Jiao et al. [19] investigated the hot deformation behavior of GH4169 alloy through compression tests at 900–1100 °C and 0.01–5 s^−1^. A constitutive model and processing map were established, revealing DDRX and CDRX as primary softening mechanisms. Optimal hot-working parameters were identified as 1000–1100 °C/0.01–0.1 s^−1^. Cui et al. [20] analyzed the hot deformation behavior of an EBSL Ni–Co superalloy via compression at 1070–1180 °C and 0.001–1 s^−1^. A strain-compensated Arrhenius model and processing map were established. Activation energies were 621.6 kJ/mol (γ + γ′) and 368.6 kJ/mol (γ). Optimal parameters were determined based on dynamic recrystallization behavior. Huang et al. [21] investigated the 650 °C tensile behavior of GH4706 Ni-based superalloy after stabilization. TEM and SIMS revealed η phase precipitation and grain-boundary elemental segregation enhanced boundary stability, reduced stress concentration, and promoted uniform plasticity, improving high-temperature ductility while slightly lowering tensile strength.

To accurately determine the forging characteristics and optimize the hot-working conditions of the GH2132 superalloy, the rational design of forging parameters is essential for achieving stable and high-quality deformation during production. In this study, isothermal compression tests were conducted on the GH2132 superalloy at deformation temperatures ranging from 850 to 1100 °C and strain rates between 0.001 and 10 s^−1^. The hot deformation behavior of the alloy was systematically investigated, and a constitutive model was established based on the Arrhenius-type hyperbolic sine function. Furthermore, three-dimensional power dissipation and hot processing maps were established to identify the optimal processing domains. These findings provide theoretical guidance for optimizing hot working parameters of the GH2132 superalloy.

## 2. Materials and Methods

**Figure 1:**
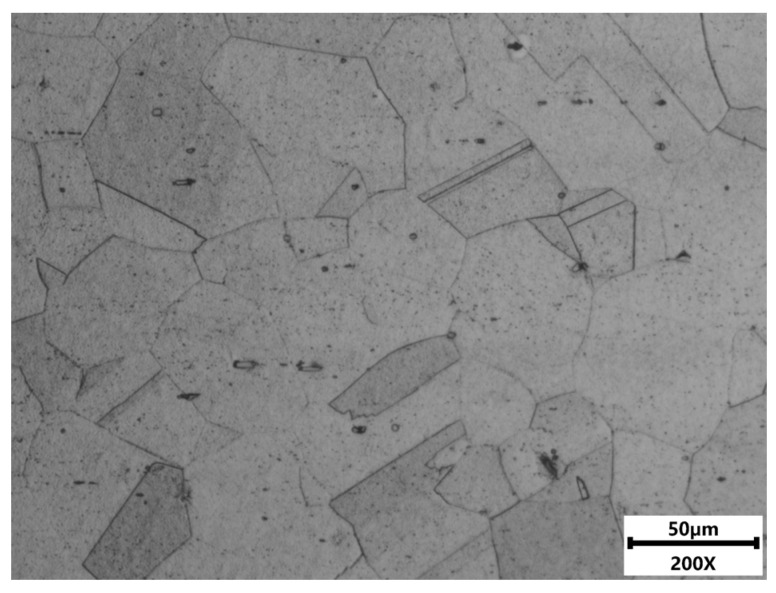
Initial microstructure of GH2132 superalloy.

The GH2132 superalloy used in this study was produced through a vacuum induction melting (VIM) followed by an electroslag remelting (ESR) process. In the VIM stage, a charge of approximately 50 kg of high-purity metals was melted in a vacuum induction furnace operated at a vacuum level not exceeding 5 × 10^−3^ Pa. The charge was subjected to low-power degassing and subsequently melted through incremental power increases within the temperature range of 1360–1420 °C. After homogenization and stirring for 25 min, the melt was cast at a cooling rate of approximately 5 °C/min to form the master alloy electrode. In the ESR stage, the master alloy was used as a consumable electrode and was remelted under 0.05 MPa Ar using a CaF_2_–CaO–Al_2_O_3_ slag system. The remelting was performed at a current of 3500–4000 A and a voltage of 45–50 V. Following purification in the slag pool, the molten alloy was solidified in a water-cooled copper mold at a cooling rate of approximately 8 °C/min to obtain a Φ80 mm ingot. The ingot was then subjected to solution treatment at 1000 °C for 1.5 h, followed by oil quenching, and aging at 700 °C for 16 h, followed by air cooling, producing the microstructural condition employed in the present study. The initial microstructure of GH2132 alloy, as shown in Figure 1 by optical microscopy, comprises equiaxed γ austenite grains of varying sizes resulting from partial recrystallization of the cast structure. Short strip or lamellar annealing twins are present within grains and along boundaries, and angular or elliptical Ti(C,N) carbonitride particles are observed within the grain regions. The corresponding chemical composition, determined by optical emission spectroscopy (OES), is summarized in Table 1.

**Figure 2:**
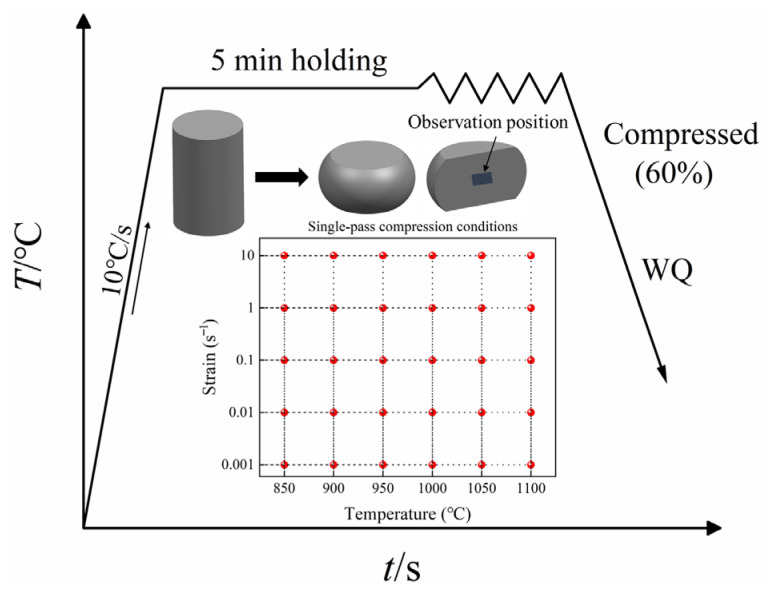
Schematic diagram of the deformation process.

Hot compression specimens with a diameter of 8 mm and a height of 12 mm were machined from the axial direction of the ingot. The tests were conducted using a Gleeble 3500-GTC thermo-mechanical simulator (Dynamic Systems Inc., Poestenkill, NY, USA). To minimize interfacial friction during deformation, graphite lubricants were applied to the specimen–anvil interfaces, and tantalum foils were inserted between the specimens and the anvils. A contact thermocouple was spot-welded at the mid-height of each specimen to ensure accurate temperature control. Before deformation, all specimens were heated at a rate of 10 °C/s and held isothermally for 5 min, allowing sufficient thermal homogenization under all deformation conditions. The deformation temperatures were set at 850, 900, 950, 1000, 1050, and 1100 °C, and the strain rates were set to 0.001, 0.01, 0.1, 1, and 10 s^−1^, resulting in 30 distinct deformation conditions. These temperature and strain rate ranges were selected to cover the typical hot working conditions of GH2132 alloy and to represent both laboratory-scale experiments and industrial forging operations. All experiments were performed under vacuum to prevent oxidation. After compression, the deformed microstructures were retained for subsequent characterization, and the true stress–strain curves were recorded. A schematic illustration of the hot compression testing procedure is provided in Figure 2.

Thin foil specimens were prepared for microstructural characterization following standard procedures. For optical microscopy, the samples were mechanically polished to a smooth surface and then etched in 10 vol.% oxalic acid for 15 s. Observations were performed using a Zeiss Axio Observer optical microscope (Carl Zeiss AG, Jena, Thuringia, Germany). For EBSD analysis, the surfaces were further refined: initial mechanical polishing using progressively finer SiC papers was followed by metallographic polishing to obtain a mirror-like finish. Electropolishing was subsequently carried out at −30 °C using an electrolyte composed of 7 wt.% perchloric acid and 93 wt.% ethanol, with a voltage of 25 V, a current density of 450 mA/cm^2^, and a duration of 1 min. Finally, Ar ion polishing was performed using a Hitachi IM4000II system (Hitachi High-Tech Corporation, Tokyo, Tokyo Metropolis, Japan) at 5 keV, 4 keV, and 3 keV sequentially, each stage conducted for 1 h, producing bright, flat surfaces with well-defined microstructural details. EBSD characterization was conducted using a Thermo Scientific Apreo 2C scanning electron microscope (Thermo Fisher Scientific, Waltham, MA, USA) equipped with an EDAX Velocity Super system (EDAX Inc., Mahwah, NJ, USA).

## 3. Results and Discussion

### 3.1. True Stress-True Strain Curves

**Figure 3:**
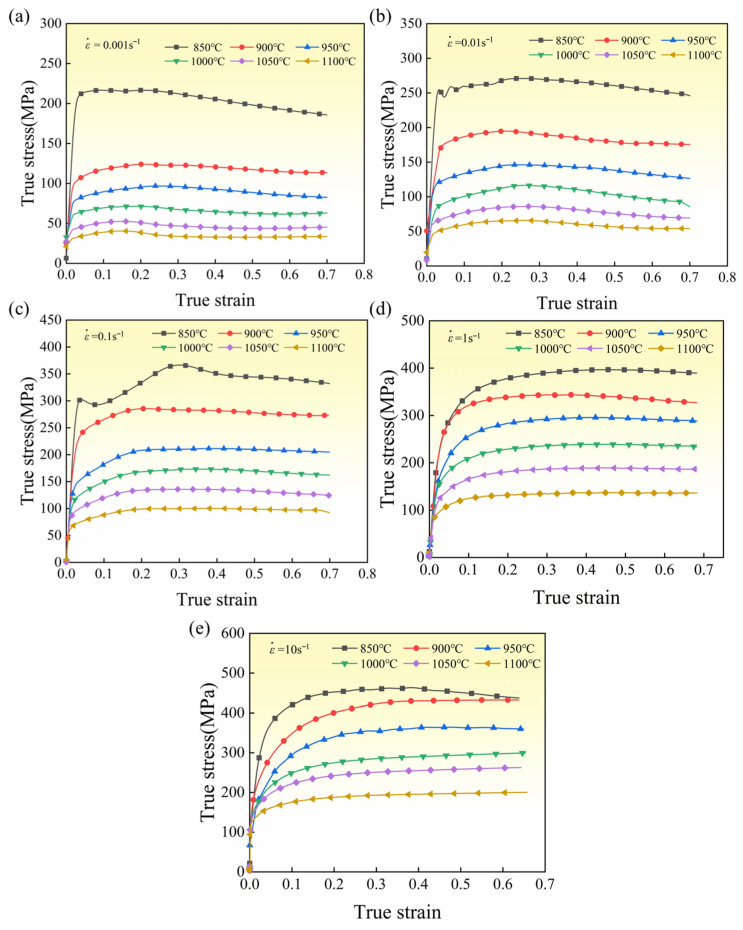
True stress–strain curves of GH2132 superalloy with different strain rates and deformation temperatures: (**a**) 0.001 s^−1^; (**b**) 0.01 s^−1^; (**c**) 0.1 s^−1^; (**d**) 1 s^−1^; (**e**) 10 s^−1^.

As shown in the stress–strain curves, both deformation temperature and strain rate exert pronounced effects on the flow behavior of the GH2132 alloy. With increasing deformation temperature, the peak flow stress exhibits a marked decline, whereas an elevation in strain rate leads to a substantial enhancement in the peak stress under identical thermal conditions. Within the investigated temperature range, the flow stress initially increases rapidly with strain, subsequently attains a maximum value, and then undergoes a gradual reduction. At a constant deformation temperature, a higher strain rate results in an overall increase in flow stress, accompanied by a shift in the peak-stress strain toward larger strain levels. As illustrated in Figure 3, the initial sharp rise in flow stress originates from intensive work-hardening mechanisms. With progressive deformation, the hardening rate diminishes, and the alloy transitions into a regime governed by uniform plastic flow. Once a balance between work hardening and dynamic recovery is established, the stress–strain response approaches a quasi-steady state. During high-temperature deformation, the alloy exhibits distinct softening behavior induced by dynamic recovery and dynamic recrystallization [22,23]. The softening associated with dynamic recovery arises from a reduction in dislocation density through dislocation glide and climb, which consequently stabilizes the flow stress [24].

### 3.2. Constitutive Modeling

The constitutive equation not only enables the prediction of the material’s flow behavior under diverse deformation conditions but also provides an effective description of its rheological response during high-temperature deformation [25,26]. In this study, the constitutive equation of the GH2132 superalloy was established based on the Arrhenius constitutive equation model [27]:

(1) $$\dot{\varepsilon }=A{\left[\sinh\left(\alpha \sigma \right)\right]}^{n}\exp\left(-Q/RT\right)$$

where $\dot{\varepsilon }$ is the strain rate, *Q* is the deformation activation energy, *n* is the stress exponent, *R* is the universal gas constant, and *A* and *α* are material constants.

When *ασ* ≤ 0.8, low-stress level:

(2) $$\dot{\varepsilon }=A_{1}\sigma^{n}\exp\left(-Q/RT\right)$$

When *ασ* ≥ 1.2, high-stress level:

(3) $$\dot{\varepsilon }=A_{2}\exp\left(\beta \sigma \right)\exp\left(-Q/RT\right)$$

For all stress conditions, the Sellars-type hyperbolic sine relationship can be expressed as:

(4) $$\dot{\varepsilon }=A{\left[\sinh\left(\alpha \sigma \right)\right]}^{n}\exp\left(-Q/RT\right)$$

Taking the natural logarithm on both sides of Equations (2)–(4), as follows:

(5) $$\ln\dot{\varepsilon }=n\ln\sigma +\ln A_{1}-\left(Q/RT\right)$$

(6) $$\ln\dot{\varepsilon }=\beta \sigma +\ln A_{2}-\left(Q/RT\right)$$

(7) $$\ln\dot{\varepsilon }=n\ln\left[\sinh\left(\alpha \sigma \right)\right]+\ln A-\left(Q/RT\right)$$

where β, *A*_1_, and *A*_2_ are constants.

**Figure 4:**
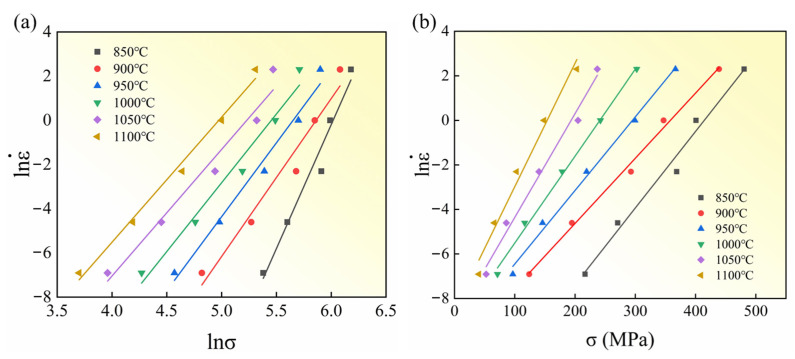
Linear relationships: (**a**) $\ln\dot{\varepsilon }$-lnσ and (**b**) $\ln\dot{\varepsilon }$-σ.

The material constants were determined by inserting the experimental data into Equations (5) and (6). Specifically, the plots of $\ln\dot{\varepsilon }$-lnσ and $\ln\dot{\varepsilon }$-σ at different temperatures are presented in Figure 4. The average slopes of the fitted lines were used to derive the values of *n* and *β*, which are 7.113 and 0.0396, respectively. According to Equation (8), the value of α was calculated as 0.00557.

(8) $$\alpha =\frac{1}{n}\frac{\partial \ln\dot{\varepsilon }}{\partial \sigma }$$

Under a specified temperature or strain rate, the integration of Equation (7) gives:

(9) $$Q=R\frac{\partial \ln\dot{\varepsilon }}{\partial \ln\left[\sinh\left(\alpha \sigma \right)\right]}{|}_{T}\frac{\partial \ln\left[\sinh\left(\alpha \sigma \right)\right]}{\partial T^{-1}}{|}_{\dot{\varepsilon }}$$

**Figure 5:**
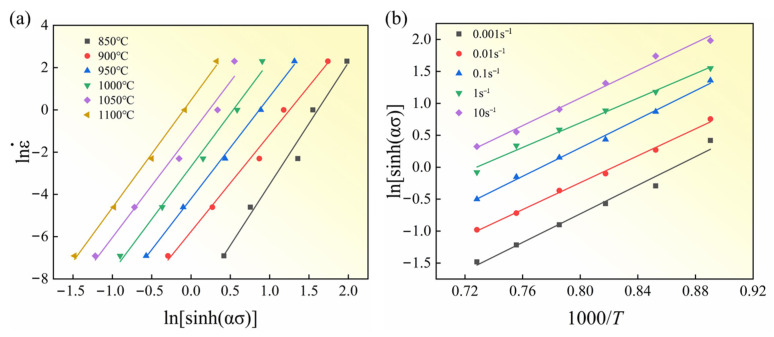
Linear relationships: (**a**) $\ln\;\dot{\varepsilon }$-ln[sinh(ασ)], (**b**) ln[sinh(ασ)]-1000/T.

The relationships between $\ln\left[\sin h\left(\alpha \sigma \right)\right]-\ln\dot{\varepsilon }$ and $\ln\left[\sin h\left(\alpha \sigma \right)\right]-1000/T$ were plotted, and as shown in Figure 5, the average slopes of $\ln\left[\sin h\left(\alpha \sigma \right)\right]-\ln\dot{\varepsilon }$ and $\ln\left[\sin h\left(\alpha \sigma \right)\right]-1000/T$ were determined to be 0.1 and 0.2, respectively. By substituting these results into Equation (9), the deformation activation energy was obtained as *Q* = 446.216 kJ/mol.

According to Equation (7), Equation (10) can be obtained as follows:

(10) $$A=\dot{\varepsilon }{\left[\sinh\left(\alpha \sigma \right)\right]}^{-n}\exp\left(Q/RT\right)$$

According to the Zener–Hollomon parameter [28], the correlation between strain rate and temperature in high-temperature deformation is expressed as follows:

(11) $$Z=\dot{\varepsilon }\exp\left(Q/RT\right)=A{\left[\sinh\left(\alpha \sigma \right)\right]}^{n}$$

*Z* represents the strain rate factor corrected for temperature. Applying the natural logarithm to both sides of Equation (11) gives:

(12) $$\ln Z=n\ln\left[\sinh\left(\alpha \sigma \right)\right]+\ln A$$

**Figure 6:**
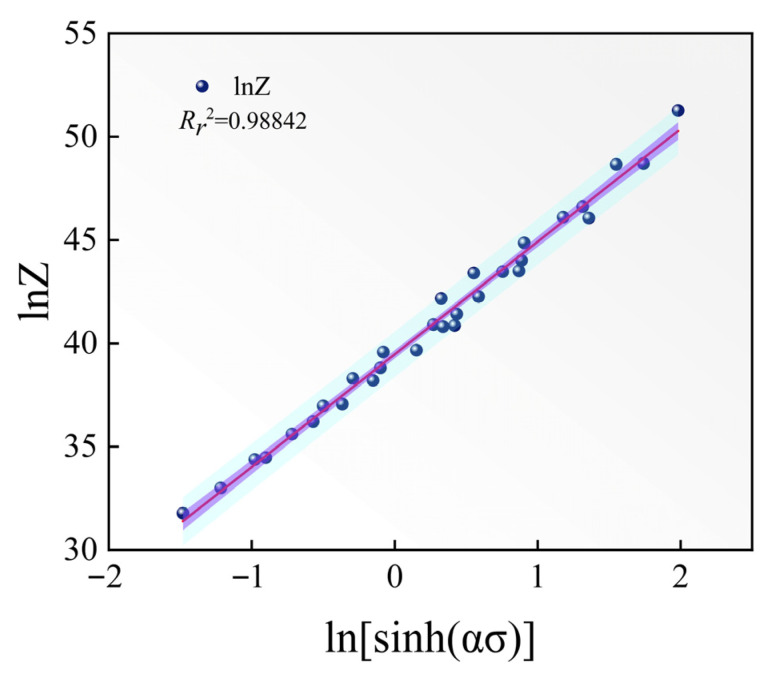
Relationships between lnZ and ln[sinh(ασ)].

The material constants were determined by inserting the peak stress values obtained under various deformation conditions into Equations (11) and (12). The correlation between ln*Z* and ln[sinh(*ασ*)] for different deformation parameters is presented in Figure 6. The fitted line produced an intercept corresponding to ln*A* = 39.48.

Based on the above calculations, the constitutive relationship model of the GH2132 superalloy was established, as expressed in Equation (13). The constitutive equation developed in this study is applicable within the temperature range of 850–1100 °C and the strain rate range of 0.001 s^−1^–10 s^−1^.

(13) $$\dot{\varepsilon }=e^{39.48}{\left[\sinh\left(0.0056\sigma \right)\right]}^{5.0303}\exp\left(-\frac{446213}{RT}\right)$$

In the formulation of the constitutive equation, the effect of strain on hot deformation behavior—which markedly influences the material constants and deformation activation energy—was not accounted for. To improve the predictive capability of the Arrhenius-type constitutive model, strain compensation is introduced by representing the material constants as polynomial functions of the true strain. Consequently, the constitutive equation connecting the flow stress with the Zener–Hollomon parameter can be formulated as [29]:

(14) $$\sigma =\frac{1}{\alpha }\ln\left\{{\left(\frac{Z}{A}\right)}^{\frac{1}{n}}+{\left[{\left(\frac{Z}{A}\right)}^{\frac{2}{n}}+1\right]}^{\frac{1}{2}}\right\}$$

**Figure 7:**
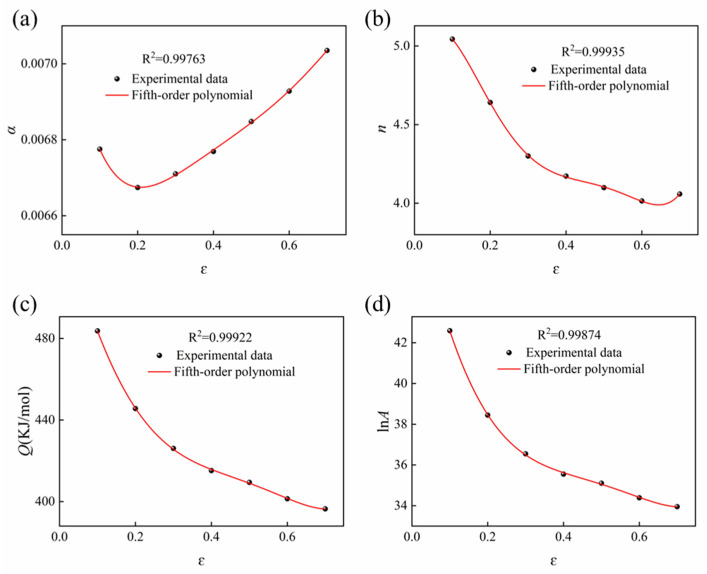
Deformation activation energy and material constants at different strain levels: (**a**) *α*-ε, (**b**) *n*-ε, (**c**) *Q*-ε, (**d**) ln*A*-ε.

For the strain-dependent constitutive modeling, a deformation interval of ε = 0.1–0.6 was selected because it corresponds to the stable plastic flow regime. At strains below 0.1, the flow stress exhibits transient fluctuations caused by rapid dislocation multiplication and short-term microstructural evolution, resulting in unstable and non-representative data. Strains exceeding 0.6 were excluded due to the onset of localized deformation and possible adiabatic heating, which may introduce additional measurement deviations. Within this physically meaningful interval, the material constants were determined at strain increments of 0.05, and the relationships between strain and the constitutive parameters (ln*A*, *α, n*, and *Q*) were well captured by a fifth-order polynomial with correlation coefficients above 0.99, as illustrated in Figure 7 and Equation (15). Lower-order polynomials could not accurately capture the nonlinear evolution of the parameters, whereas higher-order polynomials introduced spurious oscillations, particularly near the boundaries of the strain interval. The use of a fifth-order polynomial provides a smooth, continuous, and analytically tractable representation that accurately reflects the global evolution of the experimental data while avoiding artificial oscillations, thereby ensuring reliable input for subsequent constitutive modeling.

(15) $$\left\{\begin{array}{l} \ln A=49.92-92.74\varepsilon +208.18\varepsilon^{2}-131.51\varepsilon^{3}-147.28\varepsilon^{4}+163.05\varepsilon^{5}\\ \alpha =0.0072-0.00692\varepsilon +0.0324\varepsilon^{2}-0.06797\varepsilon^{3}+0.0697\varepsilon^{4}-0.0275\varepsilon^{5}\\ n=4.8959-8.0897\varepsilon -92.4986\varepsilon^{2}+304.0544\varepsilon^{3}-422.6347\varepsilon^{4}+214.2433\varepsilon^{5}\\ Q=543.45-707.7457\varepsilon +1032.6458\varepsilon^{2}+1017.5152\varepsilon^{3}-4017.613\varepsilon^{4}+2725\varepsilon \end{array}\right.$$

**Figure 8:**
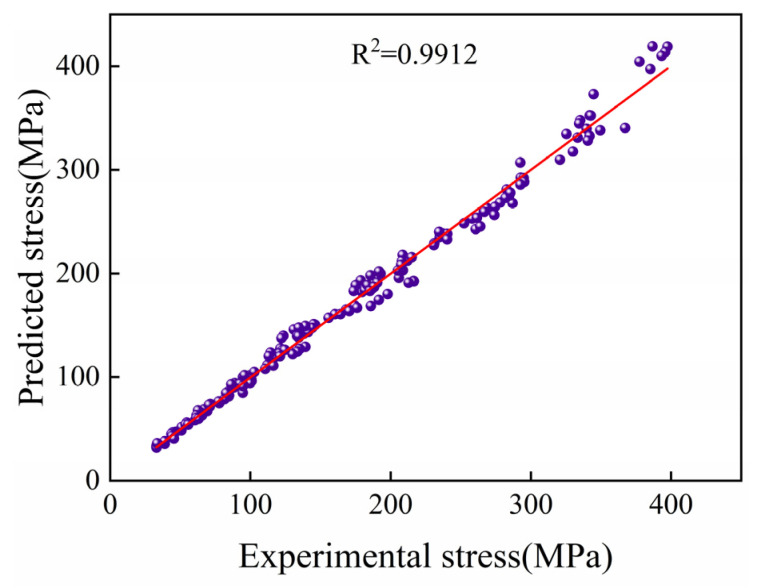
Correlation analysis of flow stress between calculated values and experimental values of GH2132 superalloy.

Figure 8 illustrates the measured and estimated flow stress values of the GH2132 superalloy using the Arrhenius-type constitutive model in different phase regions. The accuracy of the developed model was evaluated using the average absolute relative error (AARE), as defined in Equation (15). The calculated AARE value is 3.86%, indicating that the Arrhenius constitutive model developed for the GH2132 superalloy can accurately predict the flow stress behavior under hot deformation conditions.

### 3.3. Hot Processing Map

The processing map serves as a crucial tool for optimizing hot deformation conditions, providing insight into the material’s plastic deformation behavior across different temperatures, strain rates, and strains [30]. By analyzing the processing map, the safe processing domains (e.g., dynamic recrystallization regions) and flow instability regions (e.g., void formation and grain boundary cracking zones) of metallic materials can be identified, providing theoretical guidance for optimizing forging parameters and precisely controlling the microstructural evolution [31].

In this study, energy dissipation and instability maps of the GH2132 superalloy were constructed based on the Prasad-modified Dynamic Materials Model [32,33] to investigate its plastic deformation characteristics and microstructural stability under different hot deformation conditions. Each distinct domain within a hot processing map corresponds to a specific deformation mechanism, which is intrinsically linked to characteristic microstructural features and mechanical responses. During hot deformation, the total power absorbed per unit volume of the material is the combined contribution of the energy dissipated through plastic deformation and that consumed by microstructural evolution processes, as expressed by the following equation:

(16) $$P=\alpha \dot{\varepsilon }=G+J$$

where *P* is the total power input per unit volume, σ is the flow stress, and $\dot{\varepsilon }$ is the strain rate. *G* is the power dissipated as heat through plastic deformation, and *J* is the power consumed by microstructural changes.

The strain rate sensitivity coefficient, m, dictates the relationship among the absorbed power, its dissipative component, and the corresponding power dissipation fraction. The parameter η denotes the material’s power dissipation efficiency, as defined in Equations (17) and (18).

(17) $$\eta =\frac{J}{J_{\max}}=\frac{2m}{1+m}$$

(18) $$J_{\max}=\frac{P}{2}$$

The strain rate sensitivity coefficient *m* is defined as expressed in Equation (19):

(19) $$m=\frac{\partial \ln\sigma }{\partial \ln\dot{\varepsilon }}$$

Murty et al. proposed a flow instability criterion to characterize the onset of rheological instability during plastic deformation [34]:

(20) $$\xi \left(\dot{\varepsilon }\right)=\frac{\partial \ln\left(\frac{m}{m+1}\right)}{\partial \ln\dot{\varepsilon }}$$

where *ξ*($\dot{\varepsilon }$) is the flow instability parameter.

**Figure 9:**
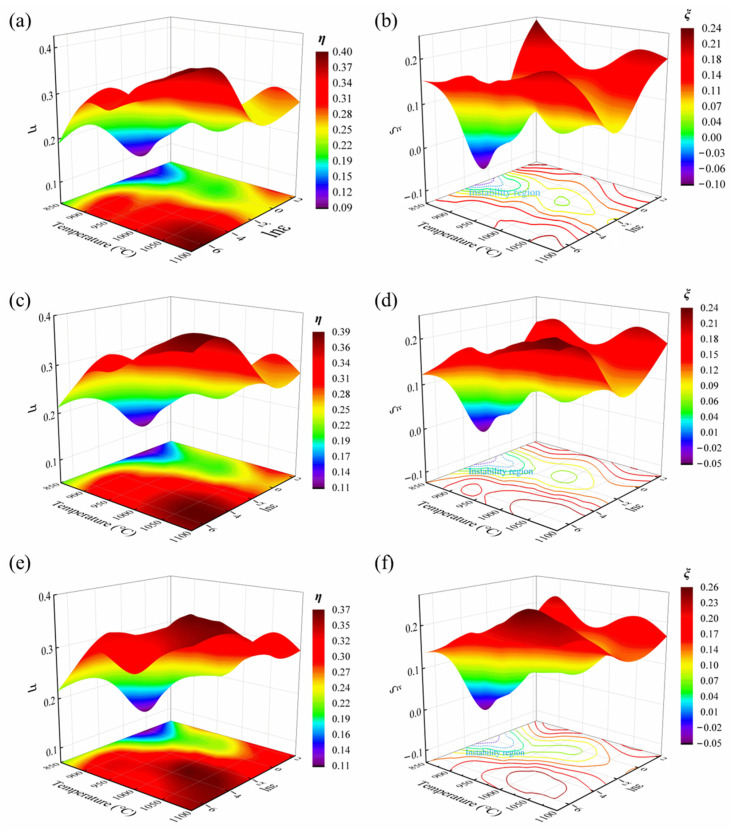
Power dissipation maps and instability maps at strain of: (**a**,**b**) 0.3, (**c**,**d**) 0.5, (**e**,**f**) 0.7.

Figure 9 illustrates the power dissipation and instability maps of the GH2132 superalloy at various true strain levels. The power dissipation map is developed based on temperature, strain rate, and the power dissipation efficiency (η). A larger η value signifies a higher fraction of energy utilized in microstructural evolution processes, including dynamic recovery and recrystallization. The instability map is developed based on temperature, strain rate, and the instability parameter *ξ*($\dot{\varepsilon }$). According to the principles of irreversible thermodynamics, *ξ*($\dot{\varepsilon }$) serves as the criterion for assessing flow instability during plastic deformation, where regions with *ξ*($\dot{\varepsilon }$) < 0 corresponds to flow instability domains.

**Figure 10:**
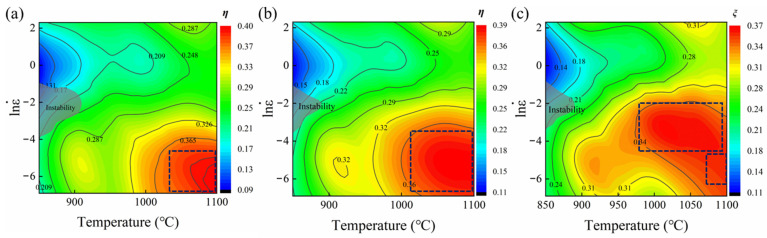
Hot processing diagrams of the tested steel at strains of (**a**) 0.3, (**b**) 0.5, and (**c**) 0.7.

The hot-processing map of the GH2132 superalloy was established by superimposing the power dissipation and instability maps. Within the safe processing domain, a higher power dissipation efficiency (*η*) reflects an enhanced propensity for dynamic recrystallization, thereby promoting flow softening and resulting in more stable deformation behavior [35,36]. In contrast, within the flow instability region, a substantial fraction of the external work is converted into plastic deformation energy, thereby decreasing the power dissipation efficiency. As a result, the material in these regions tends to develop defects such as cracking, adiabatic shear bands, and localized plastic deformation [37,38]. Figure 10 presents the hot-processing maps of the GH2132 superalloy at various true strain levels, constructed from deformation data covering the temperature range of 850–1100 °C and strain rates of 0.001–10 s^−1^. The gray-shaded regions indicate the domains of flow instability. At true strains of 0.3, 0.5, and 0.7, a distinct instability zone appears within the deformation temperature range of 850–900 °C and strain rate range of 0.01–0.1 s^−1^. Hot deformation of the GH2132 superalloy under these conditions is prone to flow instability; therefore, these processing regions should be avoided in practical manufacturing applications. The regions enclosed by the dashed boxes represent the safe processing domains for the GH2132 superalloy. Accordingly, optimal hot-working conditions for the alloy are achieved at deformation temperatures of 1000–1100 °C and strain rates of 0.001 s^−1^–0.01 s^−1^.

### 3.4. Microstructural Evolution Analysis

#### 3.4.1. Metallographic Analysis

**Figure 11:**
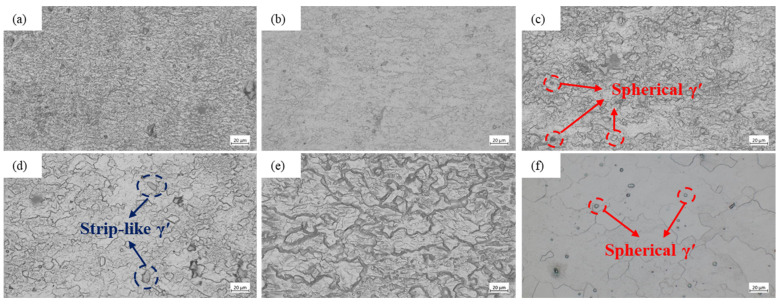
Microstructure of GH2132 Superalloy at 0.01 s^−1^: (**a**) 850 °C, (**b**) 900 °C, (**c**) 950 °C, (**d**) 1000 °C, (**e**) 1050 °C, (**f**) 1100 °C.

**Figure 12:**
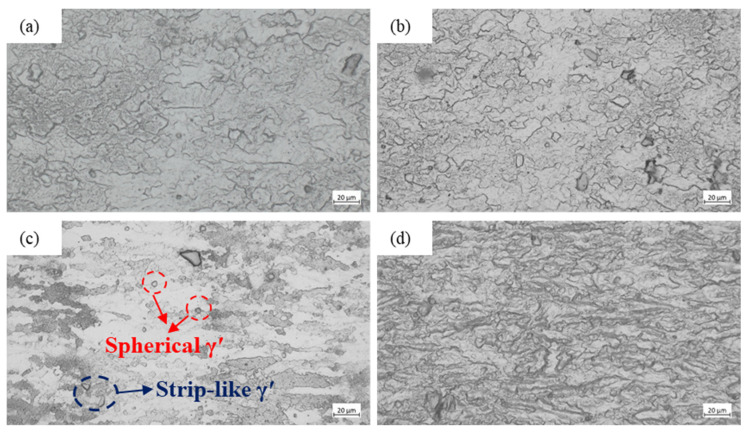
Microstructure of GH2132 Superalloy at 1000 °C: (**a**) 0.001 s^−1^, (**b**) 0.01 s^−1^, (**c**) 0.1 s^−1^, (**d**) 1 s^−1^.

The lowest temperature at which recrystallization begins to occur is defined as the recrystallization temperature of the alloy [39,40]. The microstructural characterization of the specimens after hot compression testing was conducted. The samples were sectioned by wire electrical discharge machining, mechanically ground and polished, followed by chemical etching, and subsequently examined using an optical microscope. The representative microstructures of the GH2132 superalloy deformed at various strain rates and deformation temperatures are illustrated in Figure 11 and Figure 12. Observations indicate that elevated deformation temperatures and reduced strain rates synergistically accelerate recrystallization kinetics. When the temperature is below 950 °C, recrystallization is negligible: the γ matrix contains uniformly dispersed spherical γ′ precipitates without evidence of recrystallized grains. At 950 °C, grains become elongated parallel to the deformation direction, and partial recrystallization occurs alongside the formation of a deformed γ matrix embedded with finely distributed γ′ precipitates. As the temperature increases to 1000 °C, partial recrystallization is enhanced; this process is accompanied by grain refinement and the nucleation of fine recrystallized grains, signifying the occurrence of dynamic recrystallization (DRX). At 1050 °C, complete recrystallization is achieved, yielding a homogeneous and significantly refined grain structure. At 1100 °C, the microstructure of GH2132 is dominated by extensive γ′ dissolution and fully developed dynamic recrystallization. As the γ′ solvus is exceeded, most γ′ precipitates are dissolved, with only fine residual particles dispersed within the γ matrix [41,42]. Enhanced diffusion at this temperature enables extensive recrystallization, resulting in straightened grain boundaries and substantially coarsened equiaxed γ grains. Therefore, it can be concluded that the recrystallization behavior of the GH2132 superalloy is strongly dependent on the deformation temperature and strain rate, with dynamic recrystallization acting as the predominant softening mechanism during high-temperature deformation.

#### 3.4.2. EBSD Analysis

**Figure 13:**
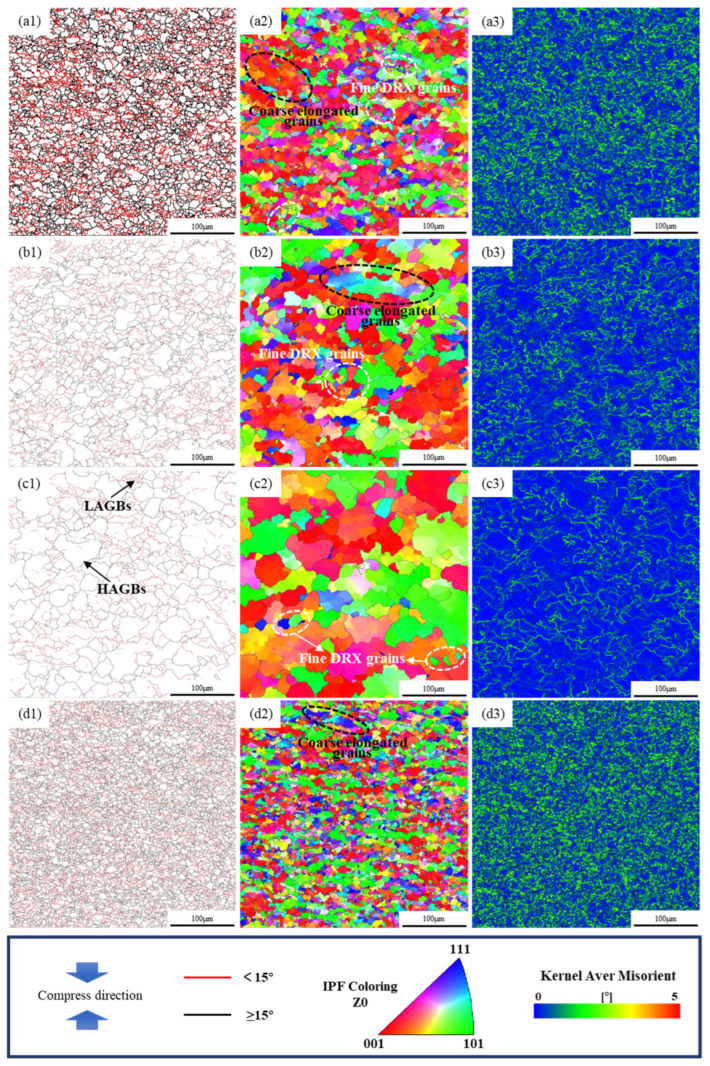
EBSD maps at different hot deformation conditions: (**a1**–**a3**) 1000 °C, 0.001 s^−1^; (**b1**–**b3**) 1050 °C, 0.001 s^−1^; (**c1**–**c3**) 1100 °C, 0.001 s^−1^; (**d1**–**d3**) 1050 °C, 0.1 s^−1^.

EBSD characterization was performed parallel to the Z_0_ direction of the compressed GH2132 alloy, wherein tests were conducted across a range of strain rates and deformation temperatures. Microstructural evolution was evaluated via grain boundary (GB) distributions, inverse pole figures (IPFs), and kernel average misorientation (KAM) maps—key metrics by which deformation and recrystallization behavior are quantified. At a strain rate of 0.001 s^−1^, a continuous transition in the microstructural response of this nickel-based superalloy was observed: this transition shifted from predominant dynamic recovery (DRV) to progressive dynamic recrystallization (DRX), as the deformation temperature was elevated from 1000 °C to 1100 °C. These combined macroscopic and microstructural characteristics allow DRV and partial DRX to be reasonably distinguished, even without explicit quantification of recrystallized nuclei. In DRV-dominated regions, the flow stress gradually reaches a plateau without a pronounced peak, and the microstructure consists of continuous subgrain structures with high orientation gradients. In contrast, partial DRX regions exhibit a stress peak followed by slight softening, accompanied by the localized formation of new grains or high-angle grain boundaries [43,44,45]. At 1000 °C (Figure 13(a1–a3)), elongated grains with irregular boundaries were observed. The EBSD orientation maps revealed pronounced subgrain structures and high orientation gradients, indicating that dynamic recovery dominated the deformation process via dislocation glide and climb, while DRX was insignificant. At 1050 °C (Figure 13(b1–b3)), noticeable boundary migration occurred, leading to the formation of fine equiaxed grains. The grain orientation became more homogeneous, with a decrease in the fraction of low-angle grain boundaries (LAGBs) and a corresponding increase in high-angle grain boundaries (HAGBs), suggesting the occurrence of partial DRX. Recrystallized grains preferentially nucleated along original grain boundaries, triple junctions, and dislocation-dense regions. When the temperature increased to 1100 °C (Figure 13(c1–c3)), the grains became coarser and more equiaxed, with smoother boundaries and a more uniform orientation distribution. High-angle boundaries dominated the misorientation maps. These features confirm that complete DRX occurred, where newly formed recrystallized grains replaced the deformed structure, resulting in full microstructural homogenization.

At the same temperature of 1050 °C, when the strain rate increased from 0.001 s^−1^ to 0.1 s^−1^ (Figure 13(d1–d3)), the grains became significantly refined and exhibited a more dispersed orientation distribution. Under a high strain rate, the deformation time is shortened, and thermal diffusion is restricted, which suppresses the growth of recrystallized grains; meanwhile, the dislocation density increases markedly, leading to the formation of dense subgrain structures. The EBSD orientation map (Figure 13(d2)) reveals a coexistence of numerous fine recrystallized grains and substructures, while the misorientation angle distribution (Figure 13(d3)) shows an increased fraction of LAGBs, indicating that the recrystallization process is incomplete and the microstructure is in a competitive stage between grain refinement and dynamic recovery. Overall, a higher temperature promotes atomic diffusion and facilitates the nucleation and growth of recrystallized grains, whereas a higher strain rate, although providing a greater stored energy (driving force), limits diffusion, resulting in a mixed microstructure characterized by high dislocation density and fine recrystallized grains.

## 4. Conclusions

This study systematically investigates the hot deformation behavior and underlying mechanisms of the GH2132 superalloy. A strain-compensated Arrhenius-type constitutive model was established to predict the flow stress of the alloy under a wide range of deformation conditions. Three-dimensional power dissipation and processing maps were constructed to further elucidate the hot working characteristics of the GH2132 superalloy. In addition, the microstructural evolution during hot compression was comprehensively analyzed using multiple characterization techniques. In industrial practice, these working windows can be used to select appropriate billet heating temperatures, deformation temperatures, and strain-rate ranges for forging or rolling schedules. Ensuring that the actual deformation path remains within these windows helps obtain uniform and refined microstructures while reducing the risk of defects such as surface cracking or excessive grain growth. These windows also provide practical constraints for finite-element simulations and process-parameter optimization. The main conclusions of this study are as follows.

(1) The peak stress of the GH2132 alloy was found to decrease significantly with increasing deformation temperature, while exhibiting a pronounced increase with rising strain rate under identical temperature conditions.
(2) An average activation energy of 446.213 kJ/mol was obtained for the deformation of the GH2132 superalloy within the deformation temperature range of 850–1100 °C and strain rate range of 0.001–10 s^−1^. The R^2^ and AARE values of the Arrhenius constitutive model are 0.9916 and 3.86%, respectively.
(3) The hot processing map of the GH2132 superalloy was constructed, and based on the Prasad flow instability criterion, the safe processing domain was identified within the deformation temperature range of 1000–1100 °C and strain rate range of 0.001–0.01 s^−1^.
(4) Microstructural analysis confirmed that the dynamic softening mechanism of the GH2132 superalloy transitions from dynamic recovery to complete dynamic recrystallization with increasing temperature and decreasing strain rate. High temperatures and low strain rates promote full recrystallization and the formation of equiaxed grains, whereas higher strain rates favor the development of refined substructures and partially recrystallized microstructures.

## Figures and Tables

**Table 1:** The chemical composition of GH2132 superalloy (wt.%).

Ni	Cr	Ti	Mn	Mo	V	Al	C	Si	B	P	S	Fe
26.85	15.71	1.96	1.24	1.04	0.37	0.36	0.027	0.08	0.0092	0.004	0.002	Bal.

## Data Availability

The original contributions presented in this study are included in the article. Further inquiries can be directed to the corresponding author.
